# Impact of Left Bundle Branch Pacing on Left Ventricular Mechanical Efficiency

**DOI:** 10.1111/jce.70387

**Published:** 2026-06-02

**Authors:** Luca Canovi, Angelo Melpignano, Michele Malagù, Cristina Balla, Ludovica Rita Vocale, Laura Rotondo, Elena Marchetti, Davide Antonio Mei, Marco Zuin, Matteo Bertini, Francesco Vitali

**Affiliations:** ^1^ Cardiology Unit, Sant'Anna University Hospital Ferrara Ferrara Italy; ^2^ Heart Rhythm Management Centre, European Reference Networks Guard‐Heart Universitair Ziekenhuis Brussel Heart Rhythm Research Brussels, Postgraduate Program in Cardiac Electrophysiology and Pacing, Vrije Universiteit Brussel Brussels Belgium; ^3^ Cardiology Unit University of Modena and Reggio‐Emilia Modena Modena Italy; ^4^ Department of Translational Medicine University of Ferrara Ferrara Italy; ^5^ Department of Cardio‐Thoraco‐Vascular Sciences and Public Health University of Padova Padua Italy

**Keywords:** cardiac pacing, conduction system pacing, left bundle branch pacing, mechanical efficiency, myocardial work, ventricular synchrony

## Abstract

**Background:**

Left bundle branch pacing (LBBP) restores physiological ventricular activation, yet its impact on left ventricular (LV) mechanical efficiency remains incompletely understood.

**Objective:**

To compare LV mechanical efficiency during spontaneous rhythm and LBBP using myocardial work (MW) indices, and to evaluate the influence of QRS transition type.

**Methods:**

In 115 patients enrolled in the prospective TREEBEARD study (NCT06324682), MW indices (i.e., global work index (GWI), global constructive work (GCW), global wasted work (GWW), and global work efficiency (GWE)) were measured before and after LBBP. Patients were classified according to QRS transition during decremental output as Left Bundle (LB) group (nonselective to selective LBB capture) or non‐LB group (nonselective capture to LV septal pacing).

**Results:**

Overall, LBBP achieved mechanical efficiency comparable to that observed during spontaneous rhythm. In the LB group, all MW indices showed strong correlations between spontaneous rhythm and LBBP. In the non‐LB group, GWI (*r* = 0.685, *p* < 0.001) and GCW (*r* = 0.750, *p* < 0.001) remained strongly correlated, whereas GWE (*r* = 0.4224, *p* = 0.01) and GWW (*r* = 0.395, *p* = 0.002) showed moderate and weak correlations, respectively.

**Conclusion:**

Based on our findings, LBBP appears to preserve LV mechanical efficiency to a degree comparable to spontaneous rhythm. Direct engagement of the conduction system, reflected by a nonselective to selective LBBP QRS transition, is associated with greater concordance of MW indices.

## Graphical abstract

The analysis of myocardial work in 115 consecutive patients receiving left bundle branch area pacing revealed a strong correlation between all myocardial work indices recorded during spontaneous or paced rhythm. Conversely, no significant correlations were observed between myocardial work indices and the R wave peak time in V6 or the V6–V1 interpeak interval. LB, left bundle; GWI, global work index; GWE, global work efficiency; GCW, global constructive work; GWW, global wasted work.

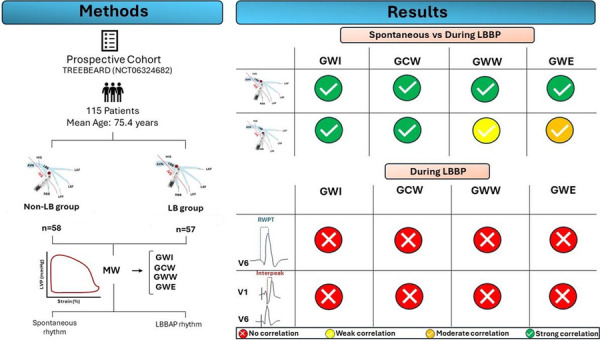

**Abbreviations**
ECG: electrocardiogram
GCW: global constructive work
GWE: global work efficiency
GWI: global work index
GWW: global wasted work
ICC: intraclass correlation
LB: left bundle
LBBP: left bundle branch pacing
LV: left ventricular
LVEF: left ventricular ejection fraction
LVSP: left ventricular septal pacing
MW: myocardial work
ns‐LBBP: nonselective left bundle branch pacing
s‐LBBP: selective left bundle branch pacing
V1RWPT: V1 R‐wave peak time
V6RWPT: V6 R‐wave peak time

## 1 Introduction

Conduction system pacing (CSP) has emerged as a physiologic alternative to conventional right ventricular pacing by preserving the native ventricular activation sequence and mitigates pacing‐induced dyssynchrony [1]. Among CSP modalities, left bundle branch pacing (LBBP) has garnered particular interest for its ability to achieve rapid and coordinated left ventricular (LV) activation. Confirmation of left bundle (LB) capture typically relies on electrocardiographic markers such as a short R‐wave peak time in lead V6 (< 75 ms), which reflects prompt recruitment of the LV conduction network [1]. However, the most definite evidence of conduction system engagement is provided by QRS morphology transitions observed during decremental output pacing [1].

Two transition patterns may occur: from nonselective (ns‐LBBP) to selective (s‐LBBP) capture, or from ns‐LBBP to left ventricular septal pacing (LVSP). During s‐LBBP, only the LB is directly captured; in ns‐LBBP, both the LB and the adjacent septal myocardium are activated simultaneously, whereas in LVSP, capture is limited to the septal myocardium. Accordingly, a transition from ns‐LBBP to s‐LBBP indicates direct engagement of the LB, while a transition from ns‐LBBP to LVSP suggests close proximity without direct conduction system capture [1].

Previous studies have demonstrated that LBBP enhances LV mechanical performance compared with LVSP [2]. Nonetheless, it remains uncertain whether direct LB engagement, evidenced by a transition from ns‐LBBP to s‐LBBP at implantation, confers additional mechanical benefits compared with transition to LVSP.

Myocardial performance depends on both electrical and mechanical efficiency. While electrical synchrony is readily demonstrated by electrocardiographic indices, mechanical efficiency can be accurately quantified by myocardial work (MW) indices derived from pressure–strain loops, a validated noninvasive surrogate of pressure–volume analysis [3, 4, 5, 6].

The present study aimed to (i) evaluate the effects of LBBP on LV mechanical efficiency compared with spontaneous rhythm using MW indices and (ii) determine whether the QRS transition pattern during implantation is associated with differences in mechanical performance.

## 2 Methods

### 2.1 Study Population and Design

We retrospectively analyzed patients who underwent LBBP at the Cardiology Unit of Azienda Ospedaliero‐Universitaria di Ferrara (Italy) between May 2023 and May 2024. All patients were part of the TREEBEARD multicentre registry (Evaluation of conTempoRary cardiac stimulation in clinical practicE: lEft, BivEntriculAr, Right, and conDuction system pacing; NCT06324682). Inclusion criteria were: (1) a Class I or IIa indication for de novo pacemaker implantation according to the current European Society of Cardiology guidelines [7]; (2) age ≥ 18 years; (3) preserved or mildly reduced LVEF (defined as a LVEF ≥ 40%); (4) successful LBBP according to the European Heart Rhythm Association consensus statement [1]; (5) evidence of of QRS morphology transition during the threshold test at implantation; (6) spontaneous rhythm before implantation without the need for temporary pacing or continuous isoprenaline infusion; and (7) written informed consent. Exclusion criteria were: inability to provide consent, pregnancy or lactation, indication for an implantable cardioverter‐defibrillator, absence of QRS transition, or LVSP criteria [1]. The study complied with the Declaration of Helsinki and approved by the local Ethics Committee (Approval Number CE‐AVEC 825/2022/Farm/AOUFe). All patients provided their written informed consent before the procedure.

### 2.2 Implantation Procedure

All procedures were performed in accordance with the European Heart Rhythm Association consensus statement [1]. Antibiotic prophylaxis and antithrombotic therapy followed institutional protocols [8, 9]. Continuous 12‐lead electrocardiogram (ECG) and intracardiac electrogram monitoring were maintained throughout the procedure. Axillary venous access was used for insertion of either lumenless or stylet‐driven pacing leads [10]. A J‐wire was used to advance the guiding catheter into the right ventricle. The catheter was then positioned against the interventricular septum under fluoroscopic guidance using a left anterior oblique (30°–40°) view, with right anterior oblique projection as needed. Counterclockwise rotation was applied to achieve perpendicular orientation to the septum, and a pace map was performed during lead exposure. Pacing threshold (0.4 ms) and impedance were measured in unipolar configuration [11].

### 2.3 Study Groups and Definitions

Immediately after lead implantation, decremental output pacing was systematically performed in unipolar configuration from the tip of the lead. Patients were categorized into two groups based on QRS morphology transitions observed during the threshold test: LB group for those with transition from ns‐LBBP to s‐LBBP; non‐LB group for those with ns‐LBBP to LVSP. ns‐LBBP to s‐LBBP transition was defined by: (i) widening of the terminal R wave in lead V1 and prolongation of the V1 R‐wave peak time (V1RWPT) > 10 ms; (ii) deeper S wave in leads V6 and I with no change in the V6 R‐wave peak time (V6RWPT); and (iii) appearance of an isoelectric interval with prolongation of the stimulus‐to‐QRS interval (measured from the pacing artifact). In contrast, ns‐LBBP to LVSP was defined by: (i) abrupt increase in V6RWPT > 10 ms; (ii) reduction in the amplitude of the R′ wave in lead V1; and (iii) attenuation or disappearance of the S wave in leads V6 and I. QRS transition was consistently confirmed intraprocedurally by two experienced operators. All measurements were performed digitally using the Sensis Vibe electrophysiology recording system (Siemens Healthineers, Erlangen, Germany). In the present study, no predefined procedural differences were adopted between patients who exhibited a transition from ns‐LBBP to s‐LBBP and those who transitioned from ns‐LBBP to LVSP. All implantations were performed according to a standardized protocol, and QRS transition patterns were assessed systematically during decremental output pacing. The ability to demonstrate selective LB capture likely reflects subtle variations in lead depth, orientation, and proximity to the left bundle fibers, as well as individual anatomical variability of the conduction system and septal substrate characteristics, rather than intentional differences in implantation strategy.

### 2.4 Echocardiographic Assessment and Myocardial Work Analysis

All patients underwent transthoracic echocardiography immediately before and within 24 h after implantation. Preimplant imaging was performed during spontaneous rhythm, whereas postimplant transthoracic imaging was recorded during pacing at a fixed rate. Pacing was programmed at 70 bpm in DDD mode with optimized atrioventricular delay for patients in sinus rhythm, or in VVI mode at 70 bpm for atrial fibrillation. Echocardiographic acquisitions were performed with a fixed ventricular output (twofold ventricular threshold or 2.5 V @ 0.4 ms) to ensure ns‐LBBP (simultaneous capture of LBB and adjacent myocardium). Blood pressure was measured before the echocardiographic examination, with the patient seated for at least 5 min, using two consecutive readings from which the mean value was calculated. Standard echocardiographic parameters were measured, including LV end‐diastolic and end‐systolic volumes, biplane left atrial volume, and LVEF, calculated by Simpson's biplane method. Transmitral flow velocities (E and A waves) were measured with the sample volume positioned at the mitral leaflet tips. Tricuspid Annular Plane Systolic Excursion was assessed according to ASE recommendations [12]. Global longitudinal strain and MW indices were derived using EchoPAC software (v202, GE Healthcare) by integrating noninvasive LV pressure curves with speckle‐tracking strain data [3, 13]. Valvular event times were used to synchronize strain and pressure data. Global work index (GWI), global work efficiency (GWE), global constructive work (GCW), and global wasted work (GWW) during spontaneous and LBBP rhythm were analyzed as indices of the MW [5, 14, 15]. In particular, GWI represents the total work performed by the left ventricle during mechanical systole (i.e., from mitral valve closure to mitral valve opening) and is quantified as the area under the LV pressure–strain loop. GCW refers to the work generated during myocardial shortening in systole combined with the negative work occurring during lengthening in isovolumetric relaxation. GWW is defined as the negative work produced during myocardial lengthening in systole plus the work generated during shortening in isovolumetric relaxation. Finally, GWE expresses the proportion of work contributing to effective ejection, calculated as the ratio of constructive work to the sum of constructive and wasted work (Table S1 and Figure S1).

### 2.5 Interobserver Variability

To assess interobserver variability, two blinded operators (L. R. V. and A. M.) performed the echocardiographic measurements and MW indices twice on the same cardiac cycles and the average values were taken. For patients with atrial fibrillation, three of the most stable cardiac cycles were identified, and measurements were performed twice for each cycle by both operators; the corresponding mean values were used for analysis. A third experienced operator was consulted in case of disagreement to reach a final decision for both ECG (F. V.) and MW indices (L. C.).

### 2.6 Study Endpoint

The endpoint was to compare GWI, GCW, GWE, and GWW between LB and non‐LB groups during spontaneous rhythm and LBBP.

### 2.7 Statistical Analysis

Data distribution was assessed using the Shapiro–Wilk test. Continuous variables are presented as mean ± standard deviation, while categorical variables are expressed as counts and percentages. Between‐group comparisons were performed using the Chi‐square or Fisher's exact test for categorical variables, and appropriate parametric or nonparametric tests for continuous variables based on data distribution. Interobserver reproducibility was evaluated using the intraclass correlation coefficient (ICC) and Bland–Altman analysis, with ICC values interpreted as follows: excellent (> 0.74), good (0.60–0.74), fair (0.40–0.59), and poor (< 0.40). Correlations between MW indices obtained during spontaneous rhythm and LBBP were assessed using Pearson's or Spearman's correlation coefficients, as appropriate. Correlations were defined as strong (*r* > 0.60), moderate (*r* = 0.40–0.59), or weak (*r* < 0.39) [16, 17]. A two‐sided *p* < 0.05 was considered statistically significant. All analyses were performed using SPSS software, version 20.0 (IBM SPSS Inc., Chicago, IL, USA).

## 3 Results

### 3.1 Study Population and Baseline Characteristics

**Figure 1:**
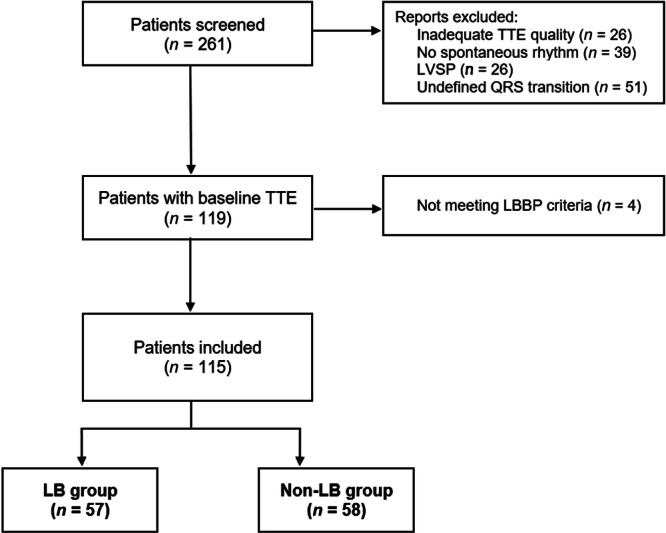
Study flowchart. LB, left bundle; LBBP, left bundle branch pacing; TTE, transthoracic echocardiography.

During the study period, 261 consecutive patients were screened, of whom 115 (mean age 75.4 ± 12.7 years; 67.8% male) met the inclusion criteria. A total of 51 patients (20%) were excluded due to undefined QRS morphology transitions. Among the study cohort, 58 patients were categorized in the non‐LB group and 57 in the LB group (Figure 1). The main pacing indications included advanced or complete atrioventricular block (80%), slow atrial fibrillation (10.4%), and sinus node disease (6.9%). No significant demographic, clinical, or echocardiographic differences were observed between the two groups (Table 1).

**Table 1:** General characteristics of the population enrolled.

	All (*N* = 115)	Non‐LB group (*n* = 58)	LB group (*n* = 57)	*p*
Age (years)	75.4 ± 12.7	74.5 ± 13.5	76.3 ± 11.8	0.46
BMI (kg/m^2^)	26.9 ± 4.9	27.4 ± 5.3	26.3 ± 4.5	0.24
Males, *n* (%)	78 (67.8)	41 (70.7)	37 (64.9)	0.50
Comorbidities				
Arterial hypertension, *n* (%)	84 (73.0)	45 (77.6)	39 (68.4)	0.26
Diabetes, *n* (%)	36 (31.1)	20 (34.5)	16 (28.1)	0.46
Smokers (current/formers), *n* (%)	40 (34.8)	20 (34.5)	20 (35.1)	0.94
Previous MI, *n* (%)	15 (13.0)	10 (17.2)	5 (8.8)	0.17
Valvular heart disease, *n* (%)	23 (20.0)	12 (20.7)	11 (19.3)	0.85
Heart failure, *n* (%)	23 (20.0)	10 (17.2)	13 (22.8)	0.45
Pacing indications				
Advanced or complete AVB, *n* (%)	92 (80)	47 (81.0)	45 (78.9)	0.78
Slow AF, *n* (%)	12 (10.4)	6 (10.3)	6 (10.5)	0.86
SND, *n* (%)	8 (6.9)	4 (6.8)	4 (7.0)	0.99
Other, *n* (%)	3 (2.6)	1 (1.7)	2 (3.5)	0.73
Baseline ECG features				
RBBB, *n* (%)	38 (33.0)	18 (31.0)	20 (35.0)	0.64
LBBB, *n* (%)	19 (16.5)	12 (20.6)	7 (12.2)	0.22
Echocardiographic parameters				
LVEF (%)	55.3 ± 7.6	55.5 ± 8.0	55.1 ± 7.2	0.83
LVEF mildly reduced (40%–50%), *n* (%)	27 (23.5)	13 (22.4)	14 (24.6)	0.72
Interventricular septum thickness (mm)	11.2 ± 1.7	10.9 ± 1.3	11.4 ± 1.9	0.13
Left atrial volume (mL/m^2^)	38.3 ± 15.1	38.2 ± 16.2	38.4 ± 13.9	0.95
TAPSE (mm)	21.3 ± 3.9	21.1 ± 3.7	21.4 ± 4.3	0.73
Global longitudinal strain (%)	−18.0 ± 2.8	−18.2 ± 2.9	−18.0 ± 2.8	0.46

Abbreviations: AF, atrial fibrillation; AVB, atrioventricular block; BMI, body mass Index; LBBB, left bundle branch block; LVEF, left ventricular ejection fraction; RBBB, right bundle branch block; SND, sinus node dysfunction; TAPSE, tricuspid annular plane systolic excursion.

### 3.2 Electrophysiological Parameters and Myocardial Work Indices

**Figure 2:**
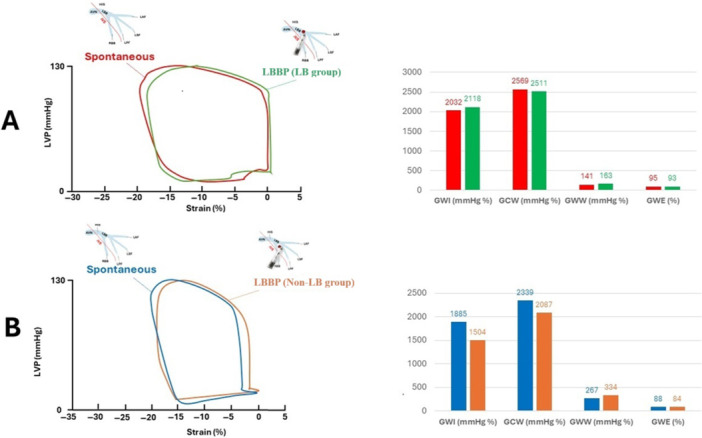
Differences in myocardial work indexes between the two study groups. (A) Example of a 64‐year‐old male included into the LB group. (B) Example of a 67‐year‐old male included into the non‐LB group.

MW indices were comparable between LB and non‐LB patients under both conditions, as well as QRS duration (Table 2 and Figure 2). Similarly, V6 R‐wave peak time (V6RWPT), and V6–V1 interpeak interval did not differ significantly between the two groups during either spontaneous rhythm or LBBP (Tables 3 and 4).

**Table 2:** ECG, pacing, and myocardial work indices.

	All (*N* = 115)	Non‐LB group (*n* = 58)	LB group (*n* = 57)	*p*
Procedure duration (min)	105.0 ± 32.6	107.6 ± 36.2	102.1 ± 28.2	0.38
*ECG*				
Spontaneous QRS duration (ms)	113.5 ± 27.3	115.0 ± 28.7	111.9 ± 25.9	0.55
LBBAP QRS duration (ms)	124.9 ± 15.0	123.8 ± 16.5	126.1 ± 13.3	0.44
V6‐RWPT (ms)	75.1 ± 9.7	76.6 ± 9.8	73.6 ± 9.6	0.10
V6–V1 interpeak interval (ms)	42.3 ± 12.4	42.2 ± 11.2	42.4 ± 13.0	0.93
Type of device implanted				
Single chamber, *n* (%)	17 (14.7)	8 (13.7)	9 (15.7)	0.76
Dual chamber, *n* (%)	98 (85.2)	50 (86.3)	48 (84.2)	0.75
*Myocardial work*				
GWI				
Spontaneous GWI (mmHg%)	1798.5 ± 348.3	1840.6 ± 316.6	1773.3 ± 379.1	0.30
LBBAP GWI (mmHg%)	1755 ± 364.2	1770.8 ± 370.9	1687.9 ± 357.4	0.23
GCW				
Spontaneous GCW (mmHg%)	2132.0 ± 355.3	2134.0 ± 327.3	2112.5 ± 342.1	0.73
LBBAP GCW (mmHg%)	2076.1 ± 407.8	2101.5 ± 419.4	2001.2 ± 389.8	0.19
GWW				
Spontaneous GWW (mmHg%)	118.2 ± 47.4	116.9 ± 50.1	119.5 ± 42.1	0.76
LBBAP GWW (mmHg%)	119.8 ± 41.6	117.9 ± 42.1	116.5 ± 42.1	0.86
GWE				
Spontaneous GWE (%)	94.0 ± 3.0	94.9 ± 2.0	94.5 ± 2.0	0.29
LBBAP GWE (%)	94.0 ± 2.0	94.5 ± 3.0	94.3 ± 1.0	0.63

Abbreviations: GCW, global constructive work; GWE, global work efficiency; GWI, global work index; GWW, global wasted work; LBBAP, left bundle branch area pacing.

**Table 3:** Relationship between myocardial indices and R‐wave peak time in lead V6 during left bundle branch area pacing, also stratified according to the direct and indirect capture of the left branch.

	V6‐RWPT vs. LBBAP GWI *r*; *p*	V6‐RWPT vs. LBBAP GCW *r*; *p*	V6‐RWPT vs. LBBAP GWW *r*; *p*	V6‐RWPT vs. LBBAP GWE *r*; *p*
All (*n* = 115)	−0.141; *p* = 0.13	−0.171; *p* = 0.67	0.001; *p* = 0.99	−0.117; *p* = 0.21
Non‐LB (*n* = 58)	−0.148; *p* = 0.26	−0.181; *p* = 0.15	0.088; *p* = 0.51	−0.162; *p* = 0.22
LB (*n* = 57)	−0.166; *p* = 0.21	−0.210; *p* = 0.11	−0.087; *p* = 0.51	−0.083; *p* = 0.53

Abbreviations: GCW, global constructive work; GWE, global work efficiency; GWI, global work index; GWW, global wasted work; LBBAP, left bundle branch area pacing.

**Table 4:** Relationship between myocardial indices and V6–V1 interpeak interval during left bundle branch area pacing, also stratified according to the direct and indirect capture of the left branch.

	V6–V1 interpeak interval vs. LBBAP GWI *r*; *p*	V6–V1 interpeak interval vs. LBBAP GCW *r*; *p*	V6–V1 interpeak interval vs. LBBAP GWW r; *p*	V6–V1 interpeak interval vs. LBBAP GWE r; *p*
All (*n* = 115)	−0.024; *p* = 0.80	−0.031; *p* = 0.74	−0.127; *p* = 0.17	−0.117; *p* = 0.21
Non‐LB (*n* = 58)	−0.013; *p* = 0.92	−0.045; *p* = 0.73	−0.220; *p* = 0.09	0.246; *p* = 0.07
LB (*n* = 57)	−0.032; *p* = 0.81	−0.101; *p* = 0.45	−0.047; *p* = 0.73	−0.006; *p* = 0.96

Abbreviations: GCW, global constructive work; GWE, global work efficiency; GWI, global work index; GWW, global wasted work; LBBAP, left bundle branch area pacing.

### 3.3 Comparison Between Spontaneous and LBBP

**Figure 3:**
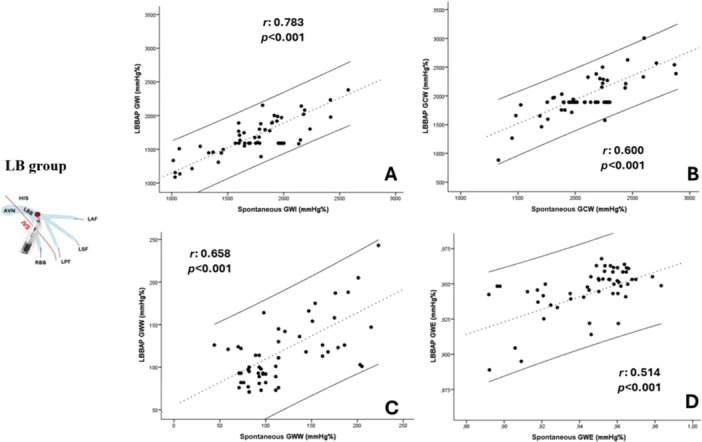
Correlation between different myocardial work indices registered during spontaneous and left bundle branch pacing in patients with direct left bundle capture. (A) Relationship between global work index registered during spontaneous and left bundle branch pacing. (B) Relationship between global constructive work registered during spontaneous and left bundle branch pacing. (C) Relationship between global wasted work registered during spontaneous and left bundle branch pacing. (D) Relationship between global work efficiency registered during spontaneous and left bundle branch pacing. GWI, global work index; GWE, global work efficiency; GCW, global constructive work; GWW, global wasted work; LBBP, left bundle branch pacing.

**Figure 4:**
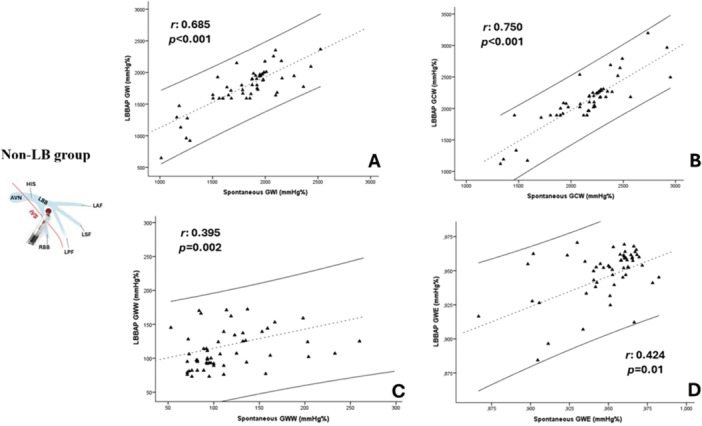
Correlation between different myocardial work indices registered during spontaneous and left bundle branch pacing in patients with indirect left bundle capture. (A) Relationship between global work index registered during spontaneous and left bundle branch pacing. (B) Relationship between global constructive work registered during spontaneous and left bundle branch pacing. (C) Relationship between global wasted work registered during spontaneous and left bundle branch pacing. (D) Relationship between global work efficiency registered during spontaneous and left bundle branch pacing. GWI, global work index; GWE, global work efficiency; GCW, global constructive work; GWW, global wasted work; LBBP, left bundle branch pacing.

Bland–Altman analysis demonstrated good interobserver reproducibility for MW indices (ICC: 0.82; 95% confidence interval: 0.61–0.96) (Figure S2). In the overall population, no significant differences were observed between spontaneous rhythm and LBBP in GWI, GCW, GWW, or GWE (Table 2). Spontaneous GWI and GCW showed strong correlations with the values measured during LBBP, whereas GWW and GWE demonstrated only moderate correlations (Table S2). In the LB group, all MW indices were significantly correlated between spontaneous rhythm and LBBP, with correlation coefficients ranging from strong to moderate: GWI (*r* = 0.737, *p* < 0.001), GCW (*r* = 0.783, *p* < 0.001), GWW (*r* = 0.600, *p* < 0.001), and GWE (*r* = 0.514, *p* < 0.001) (Figure 3A–D). In the non‐LB group, GWI (*r* = 0.685, *p* < 0.001) and GCW (*r* = 0.750, *p* < 0.001) maintained strong correlations, whereas GWE (*r* = 0.424, *p* = 0.01) and GWW (*r* = 0.395, *p* = 0.002) showed weaker, though still statistically significant, associations (Figure 4A–D). A total of 27 patients (23.5%) had a baseline LVEF < 50% (all within the mildly reduced range, as patients with LVEF < 40% were excluded by design), with a similar distribution between the non‐LB and LB groups (13 [22.4%] vs. 14 [24.6%], *p* = 0.72). In this subgroup, MW indices during LBBP were comparable to those measured during spontaneous rhythm, without significant differences between LB and non‐LB groups. Likewise, in patients with LVEF ≥ 50%, LBBP preserved LV mechanical efficiency, and no significant interaction was observed between LVEF category and QRS transition pattern.

## 4 Discussion

The main findings of this study are summarized in Central Illustration 1:

1. MW indices during spontaneous rhythm and LBBP did not significantly differ in the overall population or within either the LB or non‐LB subgroups.
2. A strong correlation between spontaneous rhythm and LBBP was observed for all MW indices in the LB group, whereas in the non‐LB group, strong correlations were limited to GWI and GCW, with GWE and GWW showing moderate and weak associations, respectively.

A previous study has demonstrated that in patients with preserved or moderately reduced LVEF, LBBP achieves LV mechanical efficiency comparable to that of spontaneous ventricular activation, whereas LVSP is associated with impaired LV mechanics and reduced GWE [3]. Moreover, LBBP has been shown to be less prone to lead microdislodgement than LVSP, a factor that may contribute to improved long‐term outcomes. This is consistent with recent data from the MELOS RELOADED trial, which reported significantly lower mortality with LBBP compared with LVSP [18].

By using MW indices as a quantitative measure of LV performance, we assessed whether QRS transition could serve as a functional surrogate of lead‐bundle interaction. Our results demonstrated no significant differences in MW indices between LB and non‐LB groups, indicating that LBBP preserves LV mechanical efficiency regardless of QRS transition pattern. These findings align with previous work from our group showing similar MW performance between His bundle pacing and LBBP, despite differences in QRS duration [15]. Collectively, these data confirm that nonselective LB capture maintains LV mechanical efficiency virtually identical to that observed during spontaneous rhythm. Patients exhibiting a transition from ns‐LBBP to s‐LBBP capture presented a strong correlation across all MW indices when compared to spontaneous rhythm. Conversely, those with a transition from ns‐LBBP to LVSP demonstrated strong correlations only for GCW and GWI, whereas GWE and GWW showed weaker associations. A possible explanation for this observation is the lower susceptibility to lead microdislodgement in the LB group [2]. In these patients, the lead tip is in direct contact with the LBB, meaning that microdislodgement is unlikely to prevent conduction system capture. In contrast, in the non‐LB group, the lead tip is often positioned adjacent to but not in contact with the LBB. Consequently, even small displacements may increase the distance from the conduction system, resulting in loss of capture and transition to LVSP.

Importantly, the preservation of LV mechanical efficiency with LBBP was consistent across patients with preserved and mildly reduced LVEF. Although the number of patients with LVEF < 50% was limited, no differential effect of QRS transition pattern was observed in this subgroup. These findings suggest that the mechanical benefits of LBBP are maintained across a spectrum of systolic function within the inclusion criteria of the present study, though larger studies including patients with more advanced LV dysfunction are needed to confirm these observations.

An additional hypothesis deserving consideration is whether the causal relationship could be reversed, namely, whether baseline mechanical inefficiency might influence the ability to achieve selective LB capture. In our cohort, MW indices measured during spontaneous rhythm, including GWW, did not differ significantly between LB and non‐LB groups, arguing against a major role of pre‐existing mechanical dysfunction in determining capture pattern. Nonetheless, subtle substrate characteristics not captured by conventional echocardiographic parameters may theoretically affect lead‐bundle interaction. Future prospective studies integrating advanced structural and functional imaging are warranted to clarify whether specific septal or mechanical phenotypes influence the likelihood of achieving direct LB capture.

Finally, our findings highlight the clinical utility of MW analysis in evaluating residual dyssynchrony and contractile performance in patients undergoing LBBP. By integrating information on both electrical activation and mechanical function, MW may help bridge the gap between timing and performance of LV contraction. Overall, MW emerges as a promising noninvasive tool to support pacing optimization and promote a more physiological pattern of LV activation in clinical practice.

## 5 Limitations

The present study has several limitations. First, its single‐center design and the relatively limited sample size represent important constraints. However, the number of enrolled patients is comparable to that reported in previous studies on this topic [2, 15]. Second, MW assessment relies on two‐dimensional speckle‐tracking echocardiography, which requires high‐quality ultrasound imaging. Consequently, patients with suboptimal acoustic windows or inadequate image quality, as well as those who were pacing‐dependent, were excluded from the analysis. Third, although decremental output pacing was systematically performed to document QRS morphology transitions, MW analysis was conducted at a fixed ventricular output to ensure stable nonselective LBB capture and standardized hemodynamic conditions. Consequently, we did not perform intrapatient comparisons of LV mechanical efficiency across different pacing morphologies (e.g., selective LBB capture or LVSP during output reduction). Therefore, potential acute mechanical differences associated with changes in capture configuration cannot be excluded. Dedicated prospective studies incorporating real‐time echocardiographic assessment during output modulation are warranted to clarify this aspect. Fourth, our study focused exclusively on instantaneous MW, without evaluating long‐term complications or clinical outcomes. Given these limitations, our findings should be considered preliminary and hypothesis‐generating. Larger multicenter studies with extended follow‐up are required to confirm the clinical impact of LBBP on hard outcomes, particularly in comparison with conventional right ventricular pacing, which has been associated with reduced MW values.

## 6 Conclusion

LBBP seems to provide LV mechanical efficiency comparable to that of spontaneous activation, regardless of whether LB capture is selective or nonselective, in patients with preserved or moderately reduced LVEF. These findings underscore the physiological advantage of engaging the LB‐Purkinje system and support LBBP as a highly effective strategy for preserving ventricular mechanics.

**Central Illustration 1:**
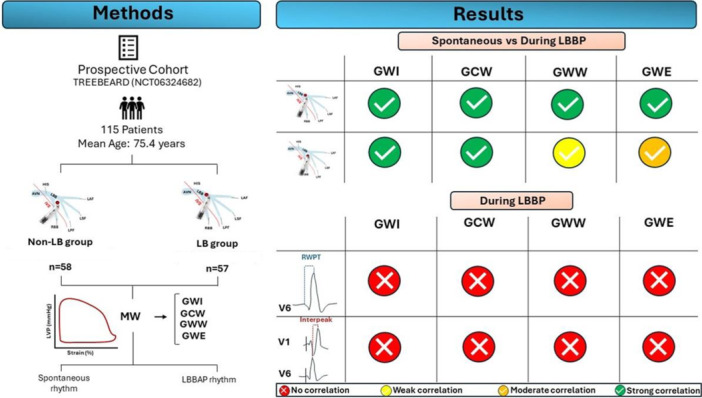
The analysis of myocardial work in 115 consecutive patients receiving left bundle branch area pacing revealed a strong correlation between all myocardial work indices recorded during spontaneous or paced rhythm. Conversely, no significant correlations were observed between myocardial work indices and the R wave peak time in V6 or the V6–V1 interpeak interval. GCW, global constructive work; GWE, global work efficiency; GWI, global work index; GWW, global wasted work; LB, left bundle.

## Ethics Statement

The study complied with the Declaration of Helsinki and approved by the local Ethics Committee (Approval Number CE‐AVEC 825/2022/Farm/AOUFe).

## Consent

All patients provided their written informed consent before the procedure.

## Conflicts of Interest

The authors declare no conflicts of interest.

## Supporting information

Supporting File

## Data Availability

The data that support the findings of this study are available from the corresponding author upon reasonable request.
