# Supplementary material for: Impact of Left Bundle Branch Pacing on Left Ventricular Mechanical Efficiency
Source: J Cardiovasc Electrophysiol. 2026 Jun 2;37(7):1554–63. doi: 10.1111/jce.70387 (PMC13372442; doi:10.1111/jce.70387)
Supplement: Supplementary file 1 — Supporting File [file JCE-37-1554-s001.docx]

**IMPACT OF LEFT BUNDLE BRANCH PACING ON LEFT VENTRICULAR MECHANICAL EFFICIENCY**

L. Canovi,^1 ,2^ MD*; A. Melpignano,^1^ MD*; M. Malagù,^1^ MD; C. Balla,^1^ MD, PhD; L.R. Vocale,^1^ MD; L. Rotondo,^1^ MD; E. Marchetti,^1^ MD; D.A. Mei,^3^ MD; M. Zuin,^1^ MD; M. Bertini,^1^ MD, PhD; F. Vitali,^1^ MD, PhD

^1^Cardiology Unit, Sant’Anna University Hospital, Ferrara, FE, Italy

^2^Heart Rhythm Management Centre, European Reference Networks Guard-Heart, Universitair Ziekenhuis Brussel Heart Rhythm Research Brussels, Postgraduate Program in Cardiac Electrophysiology and Pacing, Vrije Universiteit Brussel, Brussels, Belgium

^3^Cardiology Unit, University of Modena and Reggio-Emilia, Modena, MO, Italy

*These authors contributed equally to this work.

Corresponding author: *Matteo Bertini (brtmtt2@unife.it)*

**Supplementary Files**

[Supplementary Table 1. Definitions of different myocardial work indices. 2](#_Toc170543493)

[Supplementary Table 2: Correlation between different myocardial work indices registered during spontaneous and left bundle branch pacing. 3](#_Toc170543494)

[Supplementary Figure 1. Graphical representation of the left ventricular pressure-strain loop for the determination of different myocardial work indices. 4](#_Toc170543495)

[Supplementary Figure 2. Bland-Altman analysis for inter-observer variability for different myocardial work indices. G 5](#_Toc170543496)

| **Name** | **Abbreviation** | **Definition** |
| --- | --- | --- |
| Global Work Index (mmHg%) | GWI | Total work done by the ventricle during mechanical systole (from mitral valve closure to mitral valve opening), including isovolumetric relaxation |
| Global Constructive Work (mmHg%) | GCW | Work performed by the ventricle that contributes to left ventricular function during systole, which corresponds to the longitudinal shortening of the cardiac muscle during systole and lengthening during isovolumetric relaxation |
| Global Wasted Work (mmHg%) | GWW | Work performed by the ventricle that does not contribute to left ventricular function during systole, which correspond to the longitudinal lengthening of the myocardial fibers during systole plus shortening during isovolumetric relaxation |
| Global Work Efficiency (%) | GWE | Ratio between GCW and the cum of constructive and wasted work. |

# **Supplementary Table 1.** Definitions of different myocardial work indices.

|  | **r** | **p** |
| --- | --- | --- |
| Spontaneous GWI vs LBBP GWI (n=115) | 0.734 | <0.001 |
| Spontaneous GCW vs LBBP GCW (n=115) | 0.751 | <0.001 |
| Spontaneous GWW vs LBBP GWW (n=115) | 0.450 | <0.001 |
| Spontaneous GWE vs LBBP GWE (n=115) | 0.457 | <0.001 |

# **Supplementary Table 2: Correlation between different myocardial work indices registered during spontaneous and left bundle branch pacing.** GWI: Global work index; GWE: Global work efficiency; GCW: Global constructive work (GCW); GWW: Global wasted work; LBBP: Left Bundle Branch Pacing

**
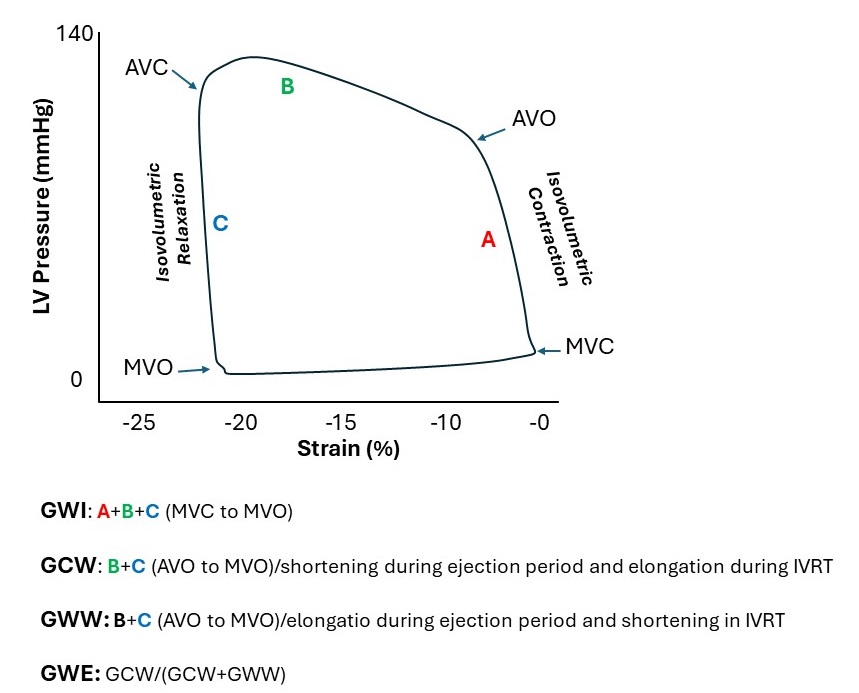
**

# **Supplementary Figure 1. Graphical representation of the left ventricular pressure-strain loop for the determination of different myocardial work indices.** AVO: Aortic valve opening; AVC: Aortic Valve Closure; MVO: Mitral valve opening; MVC: Mitral Valve Closure; GWI: Global work index; GWE: Global work efficiency; GCW: Global constructive work (GCW); GWW: Global wasted work; IVRT: Isovolumetric relaxation time.

**
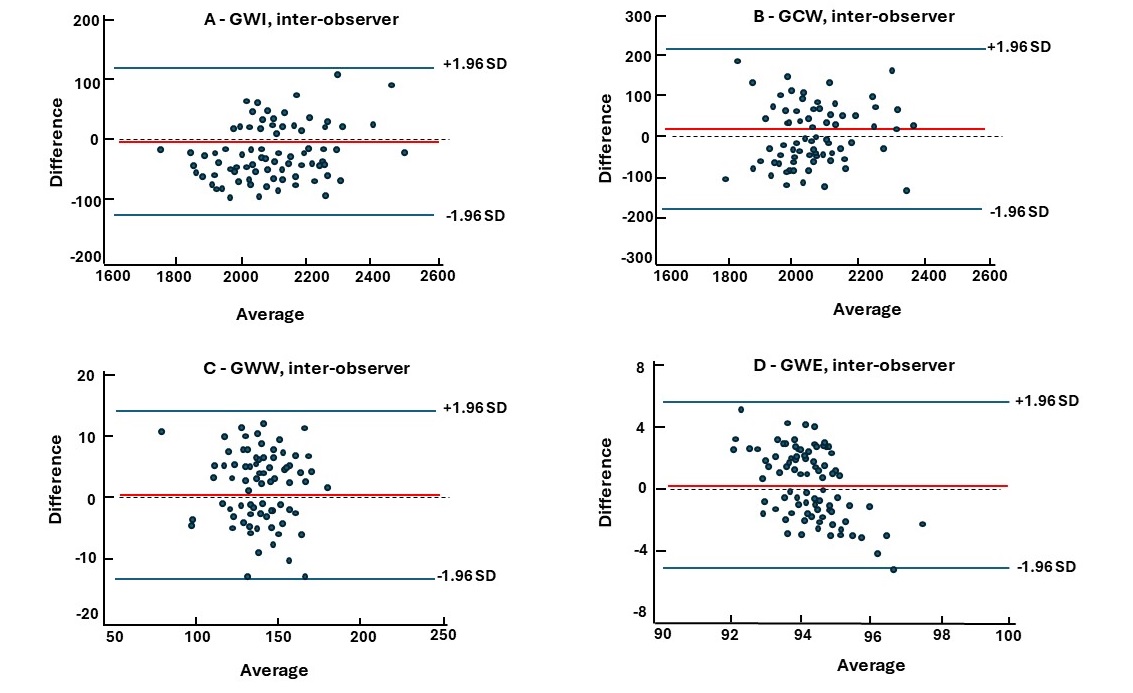
**

# **Supplementary Figure 2.** Bland-Altman analysis for inter-observer variability for different myocardial work indices. GWI: Global work index; GWE: Global work efficiency; GCW: Global constructive work (GCW); GWW: Global wasted work
